# The evolution of preferred male traits, female preference and the G matrix: “Toto, I’ve a feeling we’re not in Kansas anymore”

**DOI:** 10.1038/s41437-024-00744-8

**Published:** 2025-01-12

**Authors:** Derek A. Roff

**Affiliations:** Department of Evolution, Ecology and Organismal Biology, 2710 Life Science Bldg, University of California, Riverside, CA 92521 USA

**Keywords:** Evolution, Genetics

## Abstract

Female preference exerts selection on male traits. How such preferences affect male traits, how female preferences change and the genetic correlation between male traits and female preference were examined by an experiment in which females were either mated to males they preferred (S lines) or to males chosen at random from the population (R lines). Female preference was predicted to increase the time spent calling by males. Thirteen other song components were measured. Preference for individual traits was greatest for time spent calling(CALL), volume(VOL) and chirp rate(CHIRP) but the major contributors in the multivariate function were CALL and CHIRP, the univariate influence of VOL arising from correlations to these traits. Estimation of β, the standardized selection differential, for CALL resulting from female preference showed that it was under strong direct selection. However, contrary to prediction, CALL did not change over the course of the experiment whereas VOL, CHIRP and other song components did. Simulation of the experiment using the estimated G matrix showed that lack of change in CALL resulted from indirect genetic effects negating direct effects. Changes in song components were largely due to indirect effects. This experiment showed that female preference may exert strong selection on traits but how they respond to such selection will depend greatly upon the **G** matrix. As predicted, female preference declined in the R lines. The genetic correlations between preference and preferred traits did not decline significantly more in the R lines, suggesting correlations resulted from both linkage disequilibrium and pleiotropy.

## Introduction

Throughout the animal kingdom, females frequently exhibit preference for particular characteristics of males (Wiens and Tuschhoff 2020). Research has typically focused upon a single male trait and examined how characteristics of the female or the environment affect female preference (Roff 2015; Kelly 2018; Bentsen et al. 2006). However, female preferences may incorporate several male characters or individual females may prefer different male traits (Hedrick and Weber 1998; Bentsen et al. 2006; Roff and Fairbairn 2015; Burley et al. 2018). Thus, evolution of female preference and the preferred male traits potentially depends upon complex genetic interactions between traits both within and between the sexes. For example, males of many orthopteran species attract females acoustically with a call that can be decomposed into components such as time spent calling, volume, frequency, pulse rate etc. If one component of the call is under stabilizing preference while another is under directional preference then the resulting response of both traits to female selection of mates will depend both upon the strength of the preference for each trait, the heritabilities of the two traits and the correlation between them (Roff et al. 2017). Song components that are correlated to the preferred traits may themselves change as correlated responses to changes in the preferred traits, even if they are not directly preferred by a female.

Females of the sand cricket, *Gryllus firmus* are attracted to several components of the song of the males: the amount of time a male spends calling (Crnokrak and Roff 1995, 1998a) and at least some components of the song (Roff et al. 2017). Heritability estimates and common garden experiments have demonstrated genetic variation in time spent calling and some of the other song components (Crnokrak and Roff 1998b; Webb and Roff 1992; Roff et al. 2003). Common garden experiments have also shown geographic variation in female preference, indicating genetic variation in female preference.

Consider the following experiment in which females are presented with a choice of two males and are mated to the one they prefer. Based on the previous experiments we would predict that the time spent calling would increase. Given that song components are inherited we would also predict that these would change in accordance with female preference. In any selection experiment we need to distinguish effects due to the selection process and extraneous factors, such as genetic drift. In part this can be resolved by multiple selection lines but typically control lines in which no selection is applied are run in parallel with selection. In the present case this would consist of mating females with males at random. If female preference is genetically correlated with preferred male traits then a true control line may not be strictly possible since female preference and the male traits will co-evolve.

It is generally posited that the genetic correlation between preference and the preferred trait is generated by linkage disequilibrium rather than pleiotropy (Kirkpatrick 1987; Pomiankowski and Iwasa 1993; Mead and Arnold 2004; Radwan 2008; Kuijper et al. 2012; Hosken and Wilson 2019). The reasoning behind this is that there is no obvious functional link between the trait and the underlying neurological process that generates the preference (Bakker and Pomiankowski 1995). However, any mutation that modified genes to affect both preference and preferred trait would be favored and increase in frequency, thereby creating a pleiotropic component to the genetic correlation. In either case preference will generate selection on the trait and thus result in linkage disequilibrium. Consequently, the genetic correlation between preference and preferred trait can be a result of linkage disequilibrium and pleiotropy. Experimental detection of a covariance between preference and preferred trait appears to be quite common (63% in 43 studies, Fowler-Finn et al. 2016) but the range, 0.049–0.207, is low (Greenfield et al. 2014) as predicted if such correlations are primarily due to linkage disequilibrium (Roff and Fairbairn 2014).

A genetic correlation resulting from linkage disequilibrium will be quickly eroded by random mating. In the absence of physical linkage, the correlation will be reduced by one half at each generation (Bakker and Pominakowski 1995; Roff 1997, p73-74). If the genetic correlation is due in part to pleiotropy, then the genetic correlation will remain constant unless population size is small enough for genetic drift to be significant (Roff 1997, p73-79). These observations give us a method by which to distinguish between genetic covariance by linkage disequilibrium alone from linkage disequilibrium plus pleiotropy.

Here I report on a selection experiment on *G. firmus* consisting of two lines: in one line females are presented with a choice of two males and are mated to the one they prefer (S lines), while in another line the female is mated to a male at random (the R lines). Each line was replicated. Based on the observed female preference, time spent singing is predicted to increase in the S lines. Other song components should change towards those most preferred by the female. Theory predicts that linkage disequilibrium will be lost over generations in the R lines and at least lost at a lower rate in the S lines. Provided female preference is inherited, we would expect that over time her preference would be degraded in the R lines relative to the S lines.

## Methods

### Experimental protocols

To initiate the starting population, 73 gravid females were collected from the environs of Jacksonville, Florida. These females had already expressed their realized preference before capture and, therefore, the genetic correlation(s) between realized preference and preferred trait(s) was expressed. Females were returned to the laboratory and placed in individual cages with dishes of earth for oviposition. Sixty-one females produced offspring: these were raised in family groups of approximately 40 per group in rearing cages following the protocol described in Roff et al. (2003). Once emergence started, cages were checked at least three times a week (Monday, Wednesday, Friday). A total of 2459 adults were collected comprising 93.1% macropterous (long-winged, LW) females and 87.0% macropterous (LW) males. From these, 155 triplets, described below, were run, with each individual in a triplet drawn from a different family collected on the same day.

**Fig. 1:**
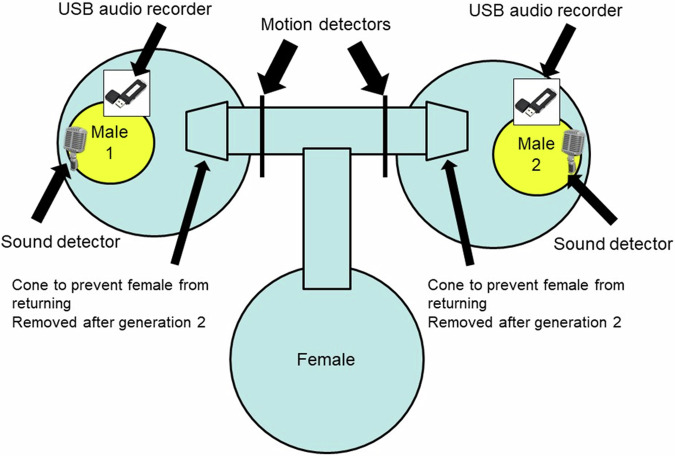
Diagram of experimental setup. In generations 1 and 2 no motion detectors were used and the T tube was set at the top of the containers, thereby preventing the female from leaving a male’s cage after she entered it. Starting at generation 3 the T tube was moved to the bottom of the cage and the cones removed, thereby permitting a female to move between cages. Motion detectors were used to monitor the female’s movement. Output from the sound detectors and motion detectors were continually sent to a recording device and stored on an SD card. Song components were recorded at generation 3 and 11 using USB audio recorders.

The initial experimental setup followed that of Crnokrak and Roff (1995). Males of a triplet were housed in individual white plastic jars (9 cm diameter) with abundant food and water. Each jar was situated in a bucket that was connected to two other buckets by plastic tubing, 2.5 cm in diameter and located just below the top of the bucket, thereby forming a T-maze. For each T-maze, two males were selected haphazardly from separate families and placed individually in the two buckets forming the top of the T. A female, also selected haphazardly from a different family, was placed in the remaining bucket that formed the bottom of the T (Fig. 1). The tubing that inter-connected all three buckets allowed the females free access to a male (a ramp in her home bucket allowed females to access the tunnels). Cones on the end of each tube leading to a male’s quarters, plus the elevated height of the tubes from the bottom of the bucket, prevented the female from exiting the male’s bucket once entered. A female could interact acoustically with a male but was visually separated by the solid wall of the male’s cage. The lid of each male cage was fitted with a wired, omnidirectional microphone (microphone name: AOM-6545P-R © PUI Audio, Inc.) by which time spent calling (CALL) was recorded. However, the microphones also recorded volume as voltage changes with an upper maximum and thus represented a truncated distribution of call volume. With the caveat that this volume measure is a truncated index I shall refer to it as call volume (VOL).

Triplets were set up in one of three growth chambers, which had shelving sufficient for 25 triplets, with each triplet being set approximately 30 cm apart and separated by thick sound reducing foam. A few preliminary tests suggested that females responded primarily to the two males of her triplet. However, there was clearly a chorus of songs in the chamber and females undoubtedly heard these and this could have affected their behavior in some way, but this would not be unrealistic given natural conditions.

With temperature regulation set in the growth chambers the fan noise was unacceptably loud. Continuous active temperature control was therefore eliminated and the daily temperature change monitored. Photoperiod was set at 15 h:9 h light:dark with lights off at 3 pm. During the dark cycle the temperature slowly fell from 30 ^o^C to 25 ^o^C, a temperature cycling that resembled that found in the natural habitat. Virtually all calling occurred during the dark cycle when the temperature was between 25–28 °C. Differences in measured components of cricket song of *Gryllus* species over this temperature range are small (*G. fultoni*, Doherty and Callos 1991, G. firmus, Pires and Hoy 1992, G. integer, Martin et al. 2011). Both males were obviously subject to the same temperature regime and hence effects of temperature differences are controlled within triplets. Male calls were recorded during an 18-h window between 3:00 p.m. (lights off) and 9:00 a.m. on the next day. This 18-h window included 9 h of dark (3:00 p.m. to midnight) and 9 h of light (midnight to 9:00 a.m.).

After testing, triplets were divided into male/female pairs based on one of two criteria. In one line, females were mated with the male in whose container she ended up, which will be referred to as the S(elected) line: two replicate lines, S1 (42 families), S2 (37 families), were so established. For the other line, named the R(andom) line, females were mated to a randomly selected male: two such replicates, R1 (47 families), R2 (39 families) were created. For subsequent generations 50 triplets per replicate were used.

Using pairs from generation 1, up to 3 cages per pair were set up with up to 40 nymphs per cage. The protocol for generation 2 followed that for generation 1. A potential problem with this setup was that females could fall into a male’s cage as a result of movements not initiated by an attraction to a song. Thus the S lines would comprise both favored males and randomly encountered males. For generation 3 and all subsequent generations the set-up was changed to monitor the movement of the females. The tubes connecting the three buckets were moved to the bottom of the containers, permitting the free movement of females to and from each male’s bucket. At the entrance to a male’s bucket was a motion detector which recorded each time the female passed into the bucket (Fig. 1). As in previous generations females still could not interact physically with the male but her attraction to a male could be evaluated by the relative number of visits to each male. The time trace was divided into 50 second bins and a male recorded as singing if detected during this period. Trips to the male were recorded as movements by the female across the motion detector towards the male. S females were mated to the males they visited most frequently, whereas R females were mated at random with a male from a different cage. Because of logistical constraints, movement of R females and measurement of song components (see below) in both S and R lines was measured only at generation 3 and 11.

From generation 3 onwards, females were still mated with the male they preferred (S lines) or at random (R lines) but their offspring (40 per pair) were produced by mass rearing in two colony cages per line (60.7 cmW × 42.6 cmD × 33 cmH). Husbandry was the same as for generations 1 and 2, except scaled up to account for the larger cage and higher number per cage. Colony cages were checked as previously: adult age was assigned as zero on the day of collecting and age of testing as the number of days since collection (individuals collected on Monday and tested the following Monday were recorded as age 7 d). In generation 3 age ranged from 3 d (1.7%) to 15 d (0.2%) with the majority (88.9%) between 7 d and 10 d. Time spent calling (CALL) is known to be related to age (Crnokrak and Roff 1998a) and so this was added to the analysis of generation 3 but in generation 11 this potentially confounding factor was avoided by all testing being done within a narrow age distribution (94.0% at 7 d or 8 d). In the closely related species, *G. pennsylvanicus*, no effect of age was detected on the song components, pulse rate, pulses per chirp, or chirp width (Ciceran et al., (1994)).

As the intent of the selection experiment was to test the effect of song selection and not wing morph (micropterous=short-winged[SW] females are significantly more fecund than macropterous[LW], Roff 1984) each female contributed 40 offspring to the next generation, giving an effective population size of 2,000. The proportion of LW individuals fluctuated from 60% to 90% but the proportions in generation 3 and 11, the generations examined in detail, were very similar. In generation 3 the combined S lines consisted of 78.4% LW females and 64.1% LW males whereas in generation 11 the combined S line consisted of 77.8% LW females and 65.0% LW males. In generation 3 the combined R lines consisted of 68.4% LW females and 59.5% LW males and in generation 11 the combined R lines comprised 68.3% LW females and 55.1% LW males. The phenotypic and genetic basis of male song characteristics and female preference was estimated using a half-sib experiment in generation 11. While this approach is able to exclude non-additive effects such as dominance and maternal effects it halves the genetic correlation between males and females established by linkage disequilibrium (Roff 1997, p73-74). Where possible, three dams per sire, were used, the dams coming from a different cage to the male: in total, CALL and VOL were measured in 2,740 male offspring from 455 families (181 sires giving an average of 2.51 dams per sire and 6 males per family). Female preference was measured in 2091 females for CALL and VOL and 1588 females for the other song components, described below.

Song characteristics of the males were obtained using usb recorders, bought from a variety of sellers through Amazon^©^ (about $10 each from China but $60 from USA outlets). In generation 3 recorders were started at the time the triplets were set up (9:30am) but most had run out of battery before recording any song, thereby considerably reducing sample size. As noted above, CALL and VOL were recorded separately from the other song components and 630 records were obtained for these components but only 223 recordings for the other components.

As calling is primarily restricted to dusk to dawn, in generation 11 the problem of run time of the recorders was alleviated by starting the recordings just before lights out (3 pm). The number successfully recorded was slightly less than for CALL and VOL: 2490 recordings spread over 444 families (179 sires, 5.6 offspring per family). The recordings were transferred to computer and scanned using the software Audacity^©^ to locate a call segment of about 2 min. This segment was then analysed using the software Raven^©^. *Gryllus firmus* is a “chirper”, meaning that its song consists of discrete chirps of generally 3–5 pulses, which preliminary analysis showed could be divided into bouts, defined as groups of chirps separated by less than 0.5 s. Songs were divided into 12 components (Fig. 1S, 14 when CALL and VOL are included): chirp rate (=number of chirps per bout=CHIRP), pulse rate (=mean pulses per chirp/mean chirp Width=PULSE), mean pulses per chirp (PULSES/CHIRP), variance in pulses per chirp (V.PULSES/CHIRP), mean chirp width (CHIRP.W), variance in chirp width (V.CHIRP.W), mean frequency (FREQ), variance in frequency (V.FREQ), mean pulse width (PULSE.W), variance in pulse width (V.PULSE.W), mean interpulse width (IPULSE.W), variance in interpulse width (V.IPULSE.W). Potentially some of these components are mathematically related. However, because within an individual male components vary, relationships can be obscured. Principal component analysis was used to determine if fewer compound components could be constructed. Overall, there was no obvious grouping that would lead to fewer variables: only 26.0% of the variation was accounted for by the first PC and nine were required to account for 93.6% of the total variation (Fig. S2). Given the lack of a clear groupings, I conducted the analyses on the original components. A similar finding was obtained for four other cricket species (Bertram et al. 2011) suggesting that the information contained in the calling song is more extensive than might be expected from such a relatively simple structure.

### Statistical analyses

#### Phenotypic analyses

Song components are measured on very different scales and were not normally distributed. To provide appropriate comparison and to better conform with the assumption of normality, each trait was transformed using the Box-Cox transformation (*boxcox* in R using *lm(x* ~ *y)*, where *x* is the data and *y* the replicate) and then standardized by subtracting the mean and dividing by the standard deviation. Thus trait values are in standard deviation units and differences among traits are comparable.

I tested for overall differences in song traits between lines using a linear mixed model with Line (S, R) and wing morph (LW, SW) as fixed effects (and where appropriate, male or female age), and replicate as a random effect. I began with a full model and then used the stepwise function *step* of R to determine the final model. For some I also used *stepAIC* which uses AIC as the metric of comparison: as both approaches gave the same qualitative result I present those from *step*. For simplicity, I present only the final model produced by this approach. Replicate was never significant and therefore the replicate lines do not appear in statistics presented: I refer to the combined lines as the S and R lines, respectively.

#### Analysis of preference

Directional preference for a particular male was measured from generation 3 onwards as the number of approaches to a focal male (arbitrarily the left male), divided by the total number of approaches to both males (Roff et al. 2017). This preference measure is estimated as D.PREF =a+bR.TRAIT, where D.PREF = (Approaches by female to focal male)/(sum of approaches to each male) and R.TRAIT =(Trait value of focal male)/(sum of Trait values of each male). In the absence of selection for preference there will likely be a decrease in the overall frequency of preference alleles and hence female realized preference is predicted to be reduced in the R lines relative to the S lines. This hypothesis leads to the prediction that the slope of the regression (*b*) in the R lines will be lower than those in the S lines.

A similar approach can be used for the case of stabilizing preference in which female preference declines as the difference between her most preferred trait value and the one encountered increases. Details of this approach are described in Roff et al. (2017) and in Supplemental materials. Theory is not a good guide to whether differences in stabilizing preference should be observed between the R and S lines. Therefore, when stabilizing preference was indicated I tested for differences between lines using a randomization test (Roff 2006), without an a priori prediction on the direction of change that might occur (see Supplemental materials).

Songs were measured at generations 3 and 11. However, as noted above, the sample size in generation 3 was small and hence the analyses presented here are from generation 11, unless otherwise specified. Female preference was first tested for each song component separately. For directional preference I included R.TRAIT (e.g. R.CALL, R.CHIRP), LINE and their interaction as fixed effects and replicate as a random effect. To construct a multivariate preference function, I used stepwise multiple regression starting with the equation

$$Y=const+LINE+\mathop{\sum }\limits_{i=1}^{9}{X}_{i}+\mathop{\sum }\limits_{i=1}^{9}LINE\ast {X}_{i}+\mathop{\sum }\limits_{j=1}^{5}{X}_{S,j}+\mathop{\sum }\limits_{j=1}^{5}LINE\ast {X}_{S,j},$$

where, for display simplicity, coefficients are omitted: *X*_i_ is the relative trait value for those nine components showing directional selection and *X*_S,j_ is the relative trait value for the five traits subject to stabilizing preference (see Results).

Prokuda (2016) showed that female preference for time spent calling is not a function of a female assessing the time a male spends calling but simply that she does not approach non-calling males. Therefore, at least some of the preference displayed by females in this experiment could be due to time when only one male was calling. Thus we would like to know female preference when both males were calling. Given that time is required to move between males such an analysis would require binning into larger bins than used to detect time spent calling (for example, say 5 minute segments). However, there is no way to assess the appropriate bin size and, therefore, I adopted an alternate approach. At R.CALL equal to 0.5 females are equally likely to select either male and thus at this value female preference will presumably be dependent on other song characteristics. To ensure an adequate sample size I chose values of R.CALL ranging from 0.47 and 0.53 and applied the above multivariate approach.

#### Genetic analysis

Heritabilities and genetic correlations were estimated in generation 11. Genetic analyses were done using the animal model as implemented by the software Asreml^©^. Line (LINE), age (AGE) and wing morph (WING) were entered as fixed effects, while sire, dam and replicate were entered as random effects. Neither age, nor replicate were statistically significant and were dropped from the final analysis. There was also no significant dam effect and thus the genetic estimates were based on sire/dam effects. For the genetic analysis it is necessary to ascribe a trait value preferred by the female: for this purpose, female preference was set as the trait value of the male she visited most. Note that is a relatively crude estimate as it is based on only two males. The estimation of the genetic correlation between male and female traits was done using the suggestion of Falconer (1952) that the two sexes be considered as different environments (Roff 1997, p89-91).

### Simulation analysis

I used the genetic parameters and female preference function estimated in generation 11 and the population data in generation 3 to predict the response to selection over the 8 generations. For a trait that occurs in both male and female the response to selection is given by Δ**z** = ½**Gβ**, where, in the present case, Δ**z** is the vector of responses, **G** is the genetic covariance matrix in standardized units and **β** is the vector of selection differentials in standardized units (Lande and Arnold 1985). An alternative writing of this equation is $\varDelta {z}_{i}=\frac{1}{2}({\beta }_{i}{h}_{i}^{2}+\mathop{\sum }\nolimits_{j=1,j\ne i}^{n-1}{\beta }_{j}{h}_{i}{h}_{j}{r}_{ij})$, where Δ*z*_*i*_ is the response in the *i*th trait, ${h}_{i}^{2}$ is the heritability of the *i*th trait, *β*_*i*_ is the standardized selection differential of the *i*th trait and *r*_ij_ is the genetic correlation between traits *i* and *j*. This version of the equation more clearly demonstrates that the response to selection is composed of a direct response, ${\beta }_{i}{h}_{i}^{2}$, and an indirect response,$\mathop{\sum }\nolimits_{j=1,j\ne i}^{n-1}{\beta }_{j}{h}_{i}{h}_{j}{r}_{ij}$, that is due to the genetic correlation between traits.

The above equations can be used to determine the response to a single round of female selection on the 14 male song traits. To most accurately mimic the experimental population, males are drawn at random from the population data in generation 3. One hundred males are drawn and paired at random, after which we use the multivariate preference function derived from the analysis of females in generation 11 to select the preferred males. Response is then calculated using the **G** matrix calculated from males in generation 11 and the **β** values generated by female preference. The mean response over *n* generations can be approximated as *n*Δz. I generated 800 responses and divided them into groups of 8 and summed the responses to give 100 simulated data sets.

To examine the effect of more than two males per female I adjusted the simulation model to allow for n males per female preference. The procedure was modeled as a sequence of encounters as follows: two males are first drawn at random from the population and presented to the female for her selection. The more preferred male is retained and another male drawn from the population at random and the female is given the choice between these two males. As before, the more preferred male is retained and another male drawn from the population. This process is continued until the female has had a choice of n males. The number of males per female was varied from 2 to 12.

## Results

### Genetic analysis of male song

LINE was a significant fixed effect in 13 of the 14 song components, whereas WING was retained in eight components, with an interaction between LINE and WING in three components (Table 1). The inclusion of WING as a fixed effect assumes a constant difference between morphs and hence a genetic correlation between wing morphs of 1: to check this I estimated the genetic correlation between wing morphs using the same approach as for the genetic correlation between the sexes. In no case is there any indication that the genetic correlation deviates significantly from 1 (Table 1).

**Table 1:** Heritability of song components, significant fixed effect differences between lines and genetic correlation between wing morphs (last three columns).

Trait	Heritability (SE)			Fixed	Genetic correlation (SE)		
	Combined	S line	R line	Effects^a^	Combined	S line	R line
CALL	0.29 (0.04)	0.27 (0.05)	0.34 (0.07)	L*W	0.82 (0.13)	0.79 (0.17)	0.86 (0.20)
VOL	0.15 (0.03)	0.11 (0.03)	0.25 (0.06)	L + W	0.74 (0.20)	0.55 (0.31)	1.00^b^
CHIRP	0.18 (0.03)	0.15 (0.04)	0.23 (0.07)	NS	1.00	1.00	0.90 (0.34)
PULSE	0.18 (0.03)	0.20 (0.04)	0.15 (0.06)	W	1.00	1.00	0.44 (0.44)
PULSE/CHIRP	0.16 (0.03)	0.18 (0.04)	0.11 (0.05)	L + W	1.00	1.00	0.93 (1.55)
V.PULSE/CHIRP	0.11 (0.03)	0.07 (0.03)	0.19 (0.07)	L	0.94 (0.35)	0.83 (0.62)	1.00
CHIRP.W	0.17 (0.03)	0.19 (0.04)	0.13 (0.06)	L	1.00	1.00	0.67 (0.96)
V.CHIRP.W	0.15 (0.03)	0.15 (0.04)	0.15 (0.06)	L	0.70 (0.24)	0.98 (0.34)	0.16 (0.44)
FREQ	0.49 (0.05)	0.53 (0.06)	0.42 (0.08)	L + W	0.90 (0.08)	0.94 (0.08)	0.73 (0.21)
V.FREQ	0.09 (0.03)	0.09 (0.03)	0.09 (0.05)	L + W	0.62 (0.48)	0.56 (0.44)	1.00
PULSE.W	0.03 (0.02)	0.02 (0.03)	0.07 (0.05)	L*W	1.00	na^c^	1.00
V.PULSE.W	0.04 (0.02)	0.04 (0.03)	0.04 (0.05)	L	0.82 (0.84)	1.00	−0.00 (1.16)
IPULSE.W	0.11 (0.03)	0.12 (0.04)	0.10 (0.05)	L*W	1.00	0.91 (0.36)	1.00
V.IPULSE.W	0.09 (0.03)	0.08 (0.03)	0.12 (0.06)	L	1.00	0.80 (0.61)	1.00

^a^L=Line, W=Wing: L*W = L + W+LxW, NS=no significant fixed effect.

^b^No SE because estimate at boundary value.

^c^No estimate because heritabilities too small for accurate calculation.

Overall, heritability was modest ($\bar{x}$ = 0.15, SE = 0.01) but significantly different from zero in 12 of the 14 song components (Table 1). No formal test of differences between the R and S lines in heritabilities was made but it is evident from the estimates that the confidence regions considerably overlap and that there are no meaningful differences (Table 1).

Genetic correlations were calculated with LINE and WING as fixed effects. Forty-nine of the 91 genetic correlations were significantly different from zero (Table 2). Genetic and phenotypic correlations were highly correlated (*r*_g_ = −0.082 + 0.853*r*_p_, *P* < 0.0001) but the variation is too large to reliably predict the genetic correlation from the phenotypic (Fig. S4). The distribution of phenotypic correlations does provide an indication of the distribution of the genetic correlations even if individual correlations cannot be predicted (histograms in Fig. S4).

**Table 2:** Genetic correlations between song components. Correlations above diagonal, SEs below the diagonal.

		1	2	3	4	5	6	7	8	9	10	11	12	13	14
1	CALL		**0.77**	**0.51**	0.21	0.16	−**0.58**	0.02	−**0.65**	**0.39**	−0.19	0.12	**0.42**	−**0.43**	−**0.66**
2	VOL	0.06		**0.57**	0.10	−0.20	−0.21	−**0.27**	−**0.46**	**0.29**	−0.06	**0.48**	**0.74**	−**0.57**	−**0.35**
3	CHIRP	0.10	0.11		0.24	−**0.35**	−0.01	−**0.48**	−0.22	**0.26**	−0.01	−0.21	0.22	−0.09	−0.05
4	PULSE	0.13	0.15	0.14		0.06	−0.18	−**0.45**	−**0.48**	0.18	0.05	−**0.57**	0.29	−**0.57**	−**0.46**
5	PULSES/CHIRP	0.12	0.13	0.13	0.14		−**0.54**	**0.84**	−**0.54**	0.04	−**0.33**	−0.09	0.12	−**0.61**	−**0.54**
6	V.PULSES/CHIRP	0.11	0.17	0.17	0.17	0.14		−**0.51**	**0.89**	−0.09	**0.50**	−**0.66**	−0.36	**0.52**	**0.72**
7	CHIRP.W	0.13	0.13	0.11	0.11	0.04	0.13		−**0.37**	−0.09	−**0.37**	0.16	−0.05	−**0.29**	−**0.31**
8	V.CHIRP.W	0.09	0.13	0.14	0.15	0.12	0.05	0.12		−**0.24**	**0.38**	−**0.57**	−**0.70**	**0.69**	**0.68**
9	FREQ	0.08	0.11	0.10	0.11	0.11	0.13	0.11	0.11		0.12	−0.17	0.17	−0.07	−0.01
10	V.FREQ	0.15	0.18	0.18	0.18	0.17	0.16	0.16	0.16	0.13		−0.31	0.16	0.21	**0.40**
11	PULSE.W	0.20	0.22	0.25	0.16	0.20	0.20	0.20	0.19	0.19	0.27		0.31	−0.39	−0.36
12	V.PULSE.W	0.21	0.24	0.24	0.23	0.20	0.30	0.21	0.24	0.18	0.27	0.36		−**0.75**	−0.06
13	IPULSE.W	0.12	0.12	0.16	0.17	0.12	0.15	0.14	0.11	0.13	0.19	0.21	0.17		**0.79**
14	V.IPULSE.W	0.11	0.16	0.17	0.21	0.15	0.10	0.15	0.11	0.14	0.18	0.24	0.29	0.13	

Estimates significantly different from zero are shown in bold.

Overall, there was no strong structure in the correlation matrix, making it difficult to predict by simple examination of the matrix of correlations how the suite of traits would evolve under multivariate female preference. However, 27 correlations exceed 0.5, indicating that responses to female preference on one or more traits will have ripple effects on the other traits.

### Effects of female preference on CALL and VOL

**Fig. 2:**
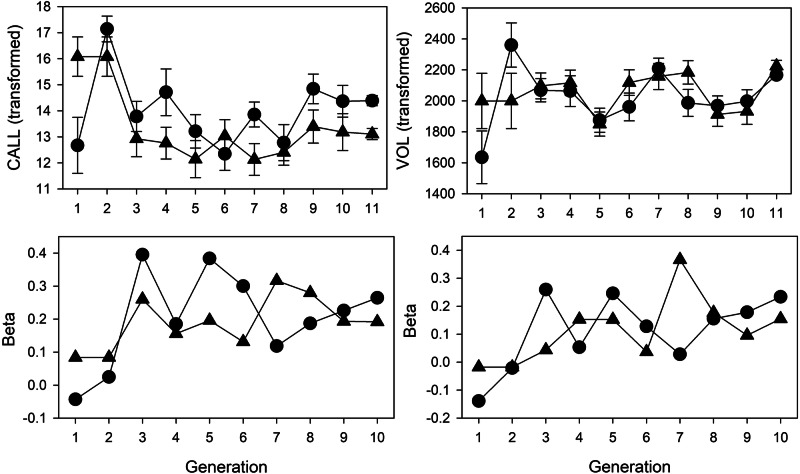
Per generation changes in CALL and VOL. Changes in CALL and VOL (top row) and selection (BETA) exerted by female preference on the two replicate S lines (bottom row).

I first focus on the principal prediction that CALL will increase in the S line and stay more or less constant in the R line. Because CALL and VOL were measured at each generation of the S lines it is possible to calculate the selection differential on these components produced by female preference (Fig. 2). Generations 1 and 2 differed from generation 3 onwards in that female choice was assessed by the container the female visited first and in which they were thereby trapped. In neither S line nor trait type (CALL, VOL) did the male selected differ significantly from the non-selected male (Selected males actually called less in generation 1 but not significantly so, *P* = 0.6388, two-tailed *t* test and called with higher volume in both lines and generations, but also not significantly so, *P* > 0.5). From generation 3 males in the S lines were chosen based on the number of times a female visited their container and in this case in both S lines **β** values were positive at generations 3 onwards for both CALL and VOL (Fig. 2). CALL of the selected males was significantly larger than the rejected males in all generations based on one-tailed *t* tests, whereas VOL was statistically significant in 13 of the 18 comparisons. The **β** values of CALL were larger, overall (mean=0.23, sd=0.08, *n* = 18), than VOL (mean = 0.15, sd = 0.09, *n* = 18).

Thus no significant selection on CALL or VOL was generated in generations 1 or 2 but significant female preference was asserted in the following generations with selection on CALL being larger than that on VOL. Despite this selection there was no clear cut change in either CALL or VOL from generation 3 to 11 (Fig. 2). Stepwise covariance analysis of CALL indicated no significant effect of generation but a significant difference between lines (*F*_3,14_ = 3.521, *P* = 0.0434). Similar analysis on VOL showed no significant effects (*F*_3,14_ = 0.165, *P* = 0.9182).

### Analysis of changes in male song components by comparing generations 3 and 11

Because of the large variation in age in generation 3, AGE was included as a fixed factor in the analysis. As we are interested in population trends WING was excluded. There were significant changes across generations in 12 of the 14 song components (Table 3). A significant response to female preference is indicated by a significant Generation by LINE interaction (G*L): such an interaction was significant in six of the song components (Table 3, Fig. S5). VOL and FREQ increased relatively more in the S lines than the R lines. In the three song components, V.PULSE/CHIRP, V.CHIRP.W and V.FREQ, S and R lines decreased over the generations but the S lines decreased less than the R lines. V.PULSE.W increased in the S lines but the R lines did not change significantly (Fig. S5).

**Table 3:** Results of stepwise regression of trait on Generation (G), Line (L), and Age (A) across generations 3 and 11.

Trait	Equation^a^	F(df1,df2)^b^	P^c^	Change over generations^d^	Female preference^e^	Simulation results^f^
VOL	G + L + G*L + A	6.13(4,3322)	<10^−5^	POS/NEG	POS	POS
FREQ	G + L + G*L	15.06(3,2655)	<10^−8^	POS	POS	POS
V.PULSES/CHIRP	G + L + G*L	18.54(3,2655)	<10^−11^	NEG	NEG	NEG
V.CHIRP.W	G + L + G*L	26.82(3,2655)	<10^−15^	NEG	NEG	NEG
V.FREQ	G + L + G*L	9.18(3,2655)	<10^−5^	NEG	STAB	NEG
V.PULSE.W	G + L + G*L	3.44(3,2655)	0.016	POS/NEG	NEG	POS
CHIRP.W	G + L	34.21(2,2656)	<10^−14^	NEG	STAB	NEG
CHIRP	G + L	6.68(2,2656)	0.001	POS	POS	POS
V.IPULSE.W	G + L	7.82(2,2656)	0.002	NEG	NEG	NEG
PULSES/CHIRP	G + L	39.15(2,2656)	<10^−15^	NEG (NS)	STAB	NEG
IPULSE.W	G + A	8.85(2,2656)	<10^−3^	NEG	NEG	NEG
CALL	L + A	11.13(2,3324)	<10^−4^	NEG(NS)	POS	POS
PULSE.W	G	13.46(1,2657)	0.001	POS	POS	NEG
PULSE	NS	na	na	POS/NEG(NS)	STAB	NEG

The table has been arranged with traits showing significant G*L interaction shown first as these are consistent with the hypothesis that these traits have responded to selection by female preference.

^a^Shows the final model structure from the stepwise analysis.

^b^F statistic for final model.

^c^Probability for final model.

^d^Change in trait value across generations. POS=trait value increased, NEG=trait value decreased, NS=not significant. Where the lines differ in their response the result for the S line is given first.

^e^See Table 3.

^f^Mean direction of change predicted by simulation model.

Time spent calling (CALL) did not change significantly from generation 3 to generation 11 (Table 3). The lack of change in CALL is consistent with the test across all generations but is surprising given that **β** was positive at each generation. In contrast, song volume (VOL) increased substantially more in the S lines (Table 6, Fig. S5), suggesting that female preference either directly or indirectly favored louder calling males, although this was not evident in the test across generations (Fig. 2).

The above results suggest that changes in song structure are a consequence of female preference. In the next section I examine this question and calculate the genetic basis of preference and the genetic correlation between female preference and male trait.

### Analysis of female preference on all song components

#### Phenotypic components of female preference as measured in generation 11

Univariate analysis of female preference showed statistical significance for all song components (12 if, instead of using *P* = 0.05 we correct for the number of tests and use a *P* of 0.05/14 = 0.0036): in five cases preference was stabilizing (Table 4, Fig. S3 shows plots for four examples of stabilizing preference), while in five cases it was directional and positive and in the remaining four cases it was directional and negative. In three cases there was a significant interaction between LINE and R.TRAIT (R.CALL, R.VOL, R.CHIRP). There was no significant difference between lines when preference was stabilizing (*P* > 0.20 in all cases). R.CALL by itself accounted for the greatest amount of variance (8.60%), followed by R.VOL (3.95%) and then R.CHIRP (3.07%), with all other song components accounting for less than 3% each. In general, the change in song traits from generation 3 to generation 11 was in the direction predicted by female preference (Table 3), though the lack of generation by line interaction makes attribution to female preference uncertain.

Theory predicted that the slope of the S lines should be steeper than that of the R lines when preference is directional: to examine this I computed the slopes of the preference regressions separately for each combined line (replicate being not significant). As predicted, the slope of the S lines was steeper in the three cases where LINE was significant (R.CALL, R.VOL, R.CHIRP) and was also true in four of the five cases where R.TRAIT was significant but LINE was not (Table 3). No prediction was made for stabilizing preference: in two cases the slope of the S lines was steeper than the R lines (R.PULSE, R.V.PULSE.W) and in three it was less steep (R.CHIRP.W, R.PULSE/CHIRP, R.V.FREQ).

**Table 4:** Results of the analysis of female preference.

Song components	Final model	Statistics for final model		Slope for lines	
		*r*^2^*100	*P*	S^d^	R
CALL	*Directional, Full*^*a*^	8.638	<10^−15^	**0.134**	0.083
VOL	*Directional, Full*	3.947	<10^−15^	**0.156**	0.081
CHIRP	*Directional, Full*	3.071	<10^−10^	**0.352**	0.157
V.IPULSE.W	Directional^b^	2.074	<10^−8^	**−0.096**	−0.042
PULSE	Stabilizing^c^	1.323	<10^−5^	**−0.068**	−0.032
CHIRP.W	Stabilizing	1.316	0.0002	−0.041	−0.052
PULSE.W	*Directional*	1.040	<10^−4^	**0.182**	0.137
IPULSE.W	*Directional*	0.920	<10^−4^	−0.104	−0.140
V.PULSE/CHIRP	*Directional*	0.902	<10^−4^	**−0.057**	−0.016
PULSE/CHIRP	Stabilizing	0.797	0.0036	−0.023	−0.041
V.CHIRP.W	*Directional*	0.709	0.0005	**−0.044**	−0.018
FREQ	*Directional*	0.693	0.0005	**0.989**	0.245
V.FREQ	Stabilizing	0.396	0.0122	−0.028	−0.048
V.PULSE.W	Stabilizing	0.316	0.0253	**−0.035**	−0.021

Traits are ranked according to variance accounted for in the final model. Directional traits in which the slope of the S line matches the direction of change from generation 3 to 11 (Fig. S5) are shown in italics.

^a^Directional, Full= R.TRAIT + LINE + R.TRAITxLINE.

^b^Directional = R.TRAIT, significant but no significant difference between lines.

^c^Stabilizing = Significant regression of *Y* on *X*, where *Y* = (Approaches by female to focal male)/(sum of approaches to each male), *X=*abs(Focal male trait-*X*_*P*_)/(sum of abs(Focal male trait- *X*_*P*_)+abs(Other male trait- *X*_*P*_)) and *X*_P_ is the value at which –*r* is maximal.

^d^Slopes in bold show cases in which the slope of the S line is steeper than that of the R line.

Stepwise analysis of the multivariate model resulted in a highly significant (*F*_11,1571_ = 16.86, *P* < 10^−15^) equation with eight additive directional terms (LINE, R.CALL, R.VOL, R.CHIRP, R.V.PULSE/CHIRP, R.V.CHIRP.W, R.IPULSE.W), one stabilizing term (PULSE, labeled as S.PULSE in the equation) and three interactions (LINExR.VOL, LINExR.CHIRP, LINExR.IPULSE.W). As the three interaction terms all involve LINE we can form two separate equations for the two lines (Table 5). In terms of magnitude, the effects of R.CALL and R.CHIRP are much greater than any other song component.

**Table 5:** Coefficients in the multivariate female preference model.

Variable	S	R	S	R
Intercept	0.332	0.508	0.270	0.179
R.CHIRP	0.234	0.059	0.354	0.354
R.CALL	0.127	0.127		
R.IPULSE.W	0.038	−0.069	0.265	0.265
R.V.CHIRP.W	0.028	0.028	−0.095	0.035
R.VOL	−0.017	−0.11		
R.V.PULSE/CHIRP	−0.025	−0.025		
S.PULSE	−0.047	−0.047	−0.145	−0.145
S.V.FREQ			0.068	0.068
S.V.PULSE.W			−0.035	0.025
V.IPULSE.W			0.025	−0.168

The three interaction terms have been absorbed into the two separate line equations and coefficients ranked according to the S line when the model using the entire data set was used. The last two columns show the results of fitting the multivariate female preference function to a reduced data set in which R.CALL is limited to the range 0.47 to 0.53.

The average time spent calling by a male over the 18-h period from 3 pm to 9am was 170.8 min (se=2.9, n = 2490, min=0, max=774.2). Males did not tend to call at the same time, one male calling by itself on average 89.0% of the time. Thus the large effect of CALL may be a consequence of solo calling. As discussed above, to test for this the data set was reduced to one in which R.CALL lay between 0.47 and 0.53. The resulting multivariate equation was highly significant (*F*_11,90_ = 3.014, *P* = 0.00184, *n* = 104) and excluded R.CALL (Table 2). R.VOL and R.V.PULSES/CHIRP, which were included in the model using the full data set were also excluded but with the reduced data set several other song components were now retained (Table 5). Particularly noteworthy is that the new equation contains three components under stabilizing preference: S.PULSE, which was previously included but now has a much larger effect (−0.047 vs −0.145), V.FREQ and V.PULSE.W.

#### Genetic components of female preference

The S and R lines differed significantly in 12 of the 14 song components (Table 6). Heritabilities for female preference were low but significant for four components (Table 6). Genetic correlations between female preference and male trait, using the combined data set were modest to high but SEs were also high and only two traits (V.CHIRP.W and IPULSE.W) were significant. The correlations estimated for the S and R lines independently are very similar (Paired t = 1.32, *P* = 0.121), though the SEs are relatively large (Table 6). If the genetic correlation is caused solely by linkage disequilibrium then, in the absence of close physical linkage, the genetic correlation in the R lines is predicted to be close to zero. The R lines do not show clearly diminished values (on average they are 0.30 greater than the S lines), suggesting that the genes for female preference and male trait are either very tightly linked or the correlations result from both linkage disequilibrium and pleiotropy.

**Table 6:** Heritability of female preference (SE) between two males and the genetic correlation (SE) between female preference and male trait.

Male trait	*h*^2^	*r*_g_^a^	*r*_g_ (S line)^b^	*r*_g_ (R line)^b^	Fixed effects	Preference type
CALL	0.03 (0.02)	0.41 (0.27)	0.30 (0.25)	1.00 (B)	LINE*SEX	Positive
VOL	0.01 (0.02)	0.39 (0.50)	−0.18 (0.67)	1.00 (B)	LINE	Positive
CHIRP	0.00 (0.00)^c^	1.00 (B)^d^	−1.00 (B)	1.00 (B)	LINE	Positive
PULSE	**0.12** (**0.04)**	0.22 (0.19)	0.22 (0.23)	0.24 (0.33)	NS	Stabilizing
PULSE/CHIRP	**0.10** (**0.04)**	0.25 (0.21)	0.26 (0.25)	0.23 (0.36)	LINE	Stabilizing
V.PULSE/CHIRP	0.05 (0.03)	0.48 (0.32)	0.35 (0.40)	0.71 (0.69)	LINE	Negative
CHIRP.W	0.04 (0.03)	0.39 (0.31)	0.43 (0.42)	0.32 (0.45)	LINE	Stabilizing
V.CHIRP.W	0.07 (0.04)	**0.82 (0.25)**	**0.88 (0.25)**	0.62 (0.63)	LINE	Negative
FREQ	**0.08** (**0.04)**	0.18 (0.18)	0.43 (0.22)	−0.35 (0.27)	LINE	Positive
V.FREQ	0.00 (0.00)	−1.00 (B)	−1.00 (B)	−0.97 (0.64)	LINE	Stabilizing
PULSE.W	0.05 (0.03)	0.55 (0.47)	0.31 (0.71)	0.83 (0.57)	LINE	Positive
V.PULSE.W	0.00 (0.03)	0.33 (1.66)	0.34 (4.98)	0.67 (1.78)	LINE	Stabilizing
IPULSE.W	**0.09** (**0.04)**	**0.85 (0.24)**	**0.79 (0.25)**	1.00 (B)	NS	Negative
V.IPULSE.W	0.03 (0.02)	1.00 (B)	1.00 (B)	1.00 (B)	LINE	Negative

Estimates using the combined data set were calculated after correcting for significant fixed effects (NS=no significant effects). Estimates in bold are significantly different from zero.

^a^Estimated using combined data set.

^b^Estimated for individual lines. Separate heritabilities not shown. As expected, they showed no clear differences.

^c^0.00 means estimate <0.005.

^d^Estimate converged on the boundary of ±1.

### Simulation model

**Fig. 3:**
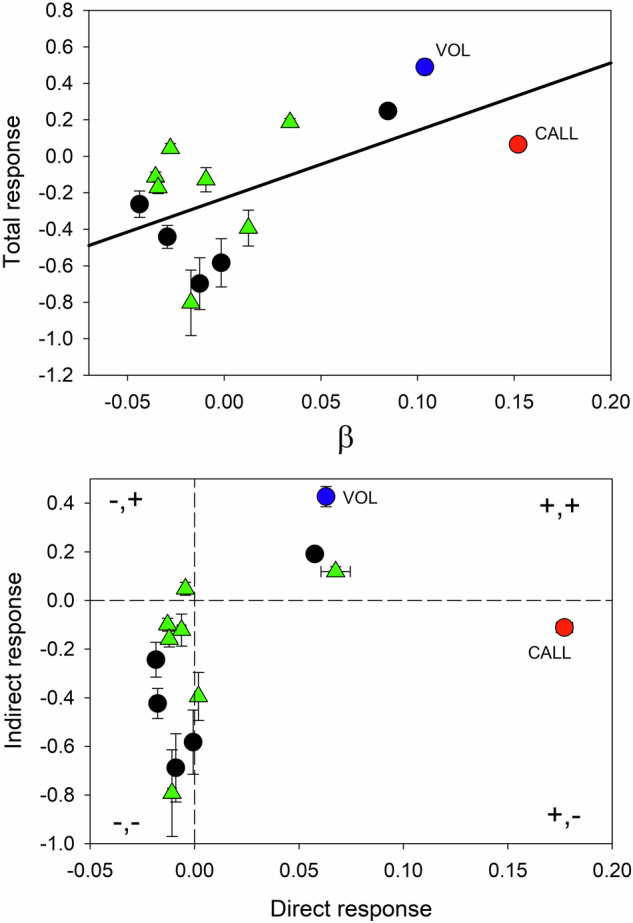
Top plot shows the mean total response (±1SE) over 8 generations for the 100 simulated experiments. Solid line shows fitted regression. Lower plot shows the division of the total response into direct and indirect responses (mean with bidirectional ±1SE). Plot divided by dotted lines into four quadrats as indicated by + and – signs (direct, indirect). Circle symbols indicate those traits comprising the multivariate female preference function. Triangle symbols show those traits not included.

Relating changes in the S lines to female preferences is made uncertain by the number of significant genetic correlations between song traits. To explore how the genetic structure can explain the observed results I used simulation. Given the uncertainty in the components of **G** (Table 2) I focus primarily on the qualitative results of the simulations. Overall, there is a significant positive correlation between the total response and **β** values (*r* = 0.594, *P* = 0.0126, one-tailed test, Fig. 3). There is no obvious distinction in response between those traits included in the female multivariate preference function and those that are not (Fig. 3). The analysis comparing generations 3 and 11 revealed significant effects of female preference for six traits: in all six cases the observed direction of the response corresponds to that predicted by the simulation (Table 3). The results for the other eight song traits are difficult to compare because the observed response is confounded by the response in the R lines; in five cases the observed change over generations is the same as that predicted by the simulation.

Indirect effects dominate all but CALL and, except for CALL, VOL and CHIRP, **β** are small, which accounts for the lack of direct effects (Table 7, Fig. 3). As expected from the multivariate female preference function and previous experiments (Crnokrak and Roff 1995, 1998) females showed a strong preference for the longer calling male, thereby generating the largest **β** value for CALL of the 14 song traits (Table 7). However, in spite of this strong direct selection the mean predicted response over 8 generations was only 0.07 sd units (sd=0.19, 31% of simulations produced negative responses, Table 7), a result that is matched by the observed insignificant response in the S lines (Table 7, Fig. 2). The apparent paradoxical result of strong selection on CALL but no response is a consequence of the large direct response (0.18) being countered by an indirect response that is almost as large in magnitude but differs in sign (−0.11, Table 7, Fig. 3). The mean **β** in the simulation is smaller than that observed in the experiment and thus might have led to the low simulated response in CALL: to test this I eliminated all **β** values less than 0.15, which then gave a mean **β** of 0.22, which was sufficiently close to the observed value of 0.23. This resulted in 52 estimates of the response over 8 generations. As expected, CALL did increase, but only to 0.08 sd units, demonstrating that it wasn’t the lowered mean **β** that resulted in the lack of response. Overall, the restriction of **β** values increased the magnitude of the trait responses (*y* = −0.001 + 1.402*x*, *r* = 0.995, *P* < 0.001, where *x* is the total response of the entire data set and *y* is the data set restricted to **β** > 0.15) but in no case the direction of change.

**Table 7:** Results of simulated response to 8 generations of female preference.

		Mean response over 8 generations			Standard errors		
Trait	beta	Total	Direct	Indirect	Total	Direct	Indirect
CALL	0.15	0.07	0.18	−0.11	0.019	0.004	0.020
VOL	0.10	0.49	0.06	0.43	0.042	0.002	0.042
CHIRP	0.08	0.25	0.06	0.19	0.024	0.002	0.024
FREQ	0.03	0.19	0.07	0.12	0.021	0.007	0.020
PULSE.W	0.01	−0.39	0.00	−0.39	0.098	0.001	0.099
IPULSE.W	0.00	−0.58	0.00	−0.58	0.132	0.002	0.132
CHIRP.W	−0.01	−0.13	−0.01	−0.12	0.066	0.002	0.066
PULSE	−0.01	−0.70	−0.01	−0.69	0.142	0.003	0.141
PULSE/CHIRP	−0.02	−0.80	−0.01	−0.79	0.179	0.002	0.179
V.PULSE.W	−0.03	0.04	0.00	0.05	0.026	0.001	0.027
V.CHIRP.W	−0.03	−0.44	−0.02	−0.42	0.063	0.002	0.062
V.IPULSE.W	−0.03	−0.17	−0.01	−0.16	0.033	0.001	0.032
V.FREQ	−0.04	−0.11	−0.01	−0.10	0.027	0.001	0.026
V.PULSE/CHIRP	−0.04	−0.26	−0.02	−0.24	0.073	0.002	0.071

**β** values are per generation values (SE < 0.004). Results have been ranked by **β** (average per generation). Sample size = 100. Response is in sd units.

Male song volume (VOL) has a negative coefficient in the multivariate preference function (Table 5) but has the second largest **β** value in the simulation (**β** = 0.10, Table 7). The large change in VOL is almost entirely due to indirect effects (direct = 0.06, indirect = 0.43, Table 7). This can be attributed in part to its positive phenotypic correlation with CALL (0.65) and CHIRP (0.25), which are the largest contributors to the preference function (Table 5). The relatively large simulated response matches the large, statistically significant observed response (Fig. S5).

**Fig. 4:**
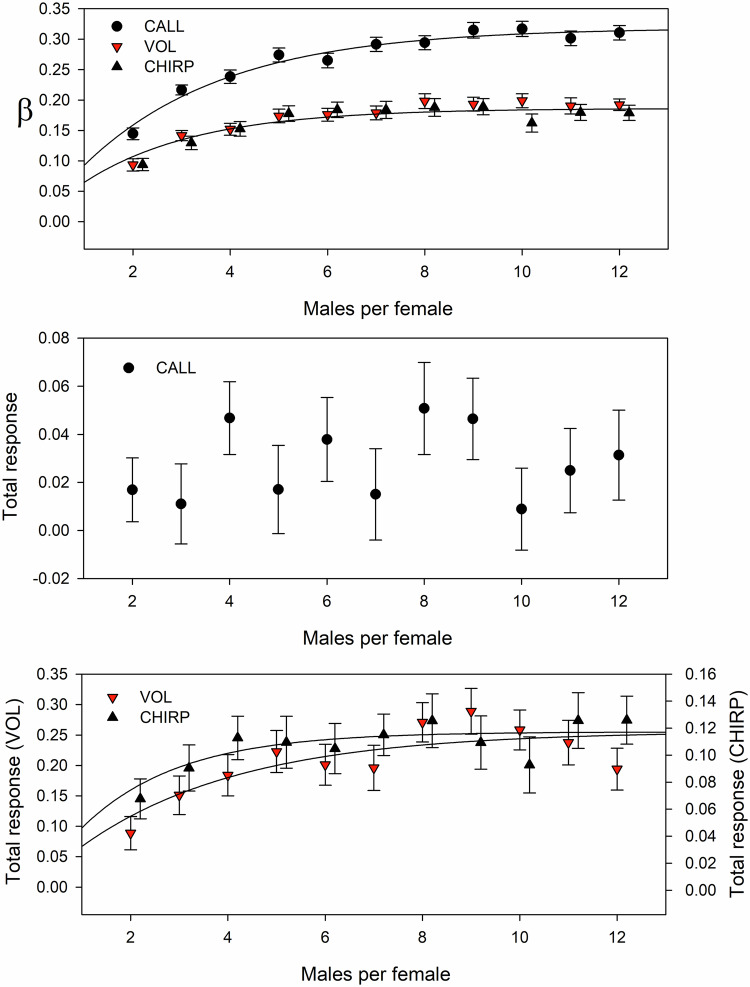
Effects on β and three traits (CALL, VOL, CHIRP) of increasing the number of males each female can evaluate. Plots show means±1SE. Curves fitted using y = a(1 − e^−bx^). For the other song traits the β function was an increasing asymptotic (FREQ, PULSE.W), a decreasing asymptotic β (V.CHIRP.W, V.FREQ, V.IPULSE.W, V.PULSE.W, V.PULSE/CHIRP), a simple decreasing function (IPULSE.W, PULSE) or showed no significant relationship (CHIRP.W, PULSE/CHIRP). This pattern was somewhat mirrored in the response of the song traits: the same general pattern was observed in VOL, CHIRP, FREQ (increasing asymptotic), V.CHIRP.W, PULSE (decreasing asymptotic), V.FREQ, V.IPULSE.W, V.PULSE/CHIRP, IPULSE.W (decreasing but not evidence of an asymptote), and CHIRP.W, PULSE/CHIRP (no significant relationship), whereas a different pattern was observed in PULSE.W (no significant relationship) and PULSE.W (decreasing asymptotic).

To examine the effect of more than 2 males per female I adjusted the simulation to vary the number from 2 to 12 (see Methods for procedure). For this analysis I examined the total response per generation with 100 replicates per male/female combination. As would be expected, **β** values increased with the number of males per female (top plot, Fig. 4) but for the three song traits subject to the highest **β** values in the 2 males/female scenario (CALL, VOL and CHIRP) the **β** values quickly levelled out at 0.32 for CALL and 0.19 for VOL and CHIRP (Fig. 4). Despite a doubling of **β** on CALL, there was no significant change in total response (Fig. 4). This lack of response is, as in the previous simulation, a consequence of direct responses being negated by the indirect responses. In contrast, in the other traits, VOL and CHIRP, the magnitude of the response increased with **β** but tended to level out at about 5–6 males/female (for VOL, *a* = 0.26 and for CHIRP, *a* = 0.12, Fig. 4).

## Discussion

Previous experiments showed that *G. firmus* females preferred males that called the most. Based on these observations I predicted that a selection experiment in which females were mated to the male that she visited the most would be the male that called more and would result in selection for longer calling males. The first prediction was supported but the second was not because the length of time spent calling (CALL) in the selected, S, lines did not increase over 11 generations of selection. Comparison with the random, R, lines also showed no change in CALL.

In addition to time spent calling, an index of call volume (VOL) was measured over all generations of selection. Females showed a positive preference for louder males but there was no change in the S lines measured over generations but there was a positive change when measured with respect to the R lines. Twelve song components were measured in generations 3 and 11 and five of these changed in the S lines relative to the R lines. Directional changes in these song components were generally consistent with directional female preference but predictions when females showed stabilizing preference were not possible by considering only the univariate situation. To address this and determine why time spent calling failed to respond as predicted I constructed a simulation model using the estimated G matrix, an estimated multivariate female preference function and initializing data from generation 3. The simulation produced results consistent, overall, with the observed changes in song components. An important finding was that while the direct response of CALL was positive, as observed, the indirect response countered this, resulting in no overall response. Further, the overall responses of other components were heavily dependent upon the indirect responses, generated by the genetic and phenotypic correlation among traits.

The simulation was modified to allow for multiple males per female still using the same preference function. CALL showed no significant response to an increase in the number of males available. Other responses were variable, though both VOL(ume) and CHIRP(rate) which previously showed the largest positive response increased asymptotically, with males per female and more or less plateaued at 5–6 males per female. The results of the simulation suggest that access to more males may not result in further selection through female preference. More males may also produce a noisy environment making discrimination difficult, thereby reducing the ability of a female to exhibit preference (Tanner and Simmons 2022).

In *G. firmus* the amount of time spent calling is a passive choice by the female in that the female does not assess the amount of time spent calling but simply does not respond to non-calling males, thereby making the male calling the most also the most conspicuous (Prokuda 2016). In the present experiment the strong response to CALL resulted from only one male calling most of the time. Eliminating the effect of CALL by restricting the female preference data to a narrow range of relative time spent calling, the resulting multivariate female preference function excluded CALL but retained chirp rate, its relative importance increasing, and six other song components, three of which were under stabilizing preference. Therefore, when two males are calling simultaneously females pay attention to the structure of their songs, particularly to chirp rate, a song characteristic also preferred in the sibling species *Gryllus pennsylvanicus* (Harrison et al. 2013). However, preference for males with a faster chirp rate will be tempered by stabilizing preference for pulse rate and to a lesser extent by stabilizing preference for variance in frequency and pulse width (Table 5). In the former case the genetic correlation is positive (0.24), permitting a reduced but positive response, but in the latter case the genetic correlations are negative (−0.01 and −0.21, respectively), acting against faster chirp rates. Such stabilizing effects of call structure have been observed in the cricket *Teleogryllus commodus* (Bentsen et al. 2006) and are probably the norm in songs of organisms that attract females by song.

Harrison et al. (2013) found that female preference increased with song volume in *G. pennsylvanicus* and that song volume correlated with body size. The univariate analysis in the present study found that female preference also increased with VOL(ume) but this was likely a result of its correlation with CALL: when CALL was controlled for, VOL(ume) was not retained in the multivariate preference function.

The importance of CALL in determining female preference will diminish as the number of males within the hearing range of the female increases. The probability of two males calling together at some given moment is *p*^2^, where *p* is the probability of a single male calling, which in the present experiment can be estimated as *p* = √0.05 = 0.224. Given *n* males, the probability of at least two males calling at any moment, provided calling does not initiate calling by other males, is determined using binomial probability, where *p* = 0.224. Niemelä et al. (2021) found that male *Gryllus campestris* were typically associated with 5 males (range 0–10). For the sequence *n* = 2, 4, 6, 8, 10 we have that the probability of at least two males calling is 0.05, 0.22, 0.40, 0.56, 0.69, or only one male calling as 0.95, 0.78, 0.60, 0.44, 0.31. Thus even with 10 males there is a significant probability that a female will hear only a single male calling and make her approach to that male, even if her preference may be greater for another of the 10 males.

Because random mating disrupts female preference, the R lines were predicted to show a reduced preference, which they did. The two song components most preferred by females, CALL and CHIRP(rate), had very low heritabilities of female preference (0.03 and <0.005, respectively, Table 6), while PULSE(rate), the third most preferred trait had the largest heritability (0.12). Using QTL analysis of *Gryllus texensis* and *G. rubens*, Blankers et al. (2019) obtained heritabilities for female preference for pulse rate of 0.61. Estimates from other organisms based on the male trait the female preferred in a choice trial are somewhat larger than obtained here for *G. firmus* (mean= 0.25, see Table 1 in Roff and Fairbairn 2015) but vary widely, ranging from 0 to 1.02 (sd=0.27).

If a female has access to all the males in the population, female preference is set by the male for whom she shows the greatest preference, which I shall call population preference. This situation will rarely be realized and I therefore define another parameter, realized preference, as her preference in a subset of males in the population. Field estimates of the number of males surveyed by females averaged only 4.5, with a median of 2.9 (Roff and Fairbairn 2014). Thus it is heritability of realized preference that is the most pertinent parameter for the study of the evolution of preference and preferred traits. As the number of males a female has access to increases, the heritability of realized preference approaches the heritability of the population preference (Roff and Fairbairn 2015) and in the present case could be as high as 0.25 (see Fig. 1 in Roff and Fairbairn 2015).

If the genetic correlation between preference and preferred trait was due entirely to linkage disequilibrium then, unless there was very tight physical linkage, in the R lines it should have diminished to zero by generation 11. Genetic coupling has been observed between male song pulse rate and female preference in *Laupala* crickets (Xu and Shaw 2019, 2021). The large number of chromosomes in *Gryllus* species, (28, Lim et al. 1973; Zefa 1999; Yoshimura 2005; Yoshimura et al. 2006) relative to Laupala, (7 autosomal linkage groups, Blankers et al. 2018), make such tight linkage less likely. Some physical linkage was observed between pulse rate and preference in QTL analysis of *G. texensis* and *G. rubens* (Blankers et al. 2019).

The genetic correlation would tend to be maintained if it were generated in part by pleiotropy. Genetic correlation estimates were typically high but, despite the large sample size, the SEs were also high, a problem that plagues such estimates (Sharma et al. 2016). Never-the-less the lack of difference between the R and S lines suggests that pleiotropy in addition to linkage disequilibrium underlies the genetic correlation in this population.

The overall finding of the experiment reported here is that a focus on a univariate female preference function or even a phenotypic examination of multivariate female preference may be quite inadequate to predict how a suite of traits preferred by a female or genetically correlated with such traits will evolve. Given even a reduced choice of males, female preference may still exert strong selection on traits but how they respond to such selection will depend greatly upon the details of the **G** matrix. In the movie “The Wizard of Oz”, Dorothy lives in Kansas, which is portrayed as a plain, gray country. She is transported to Oz, which, by comparison, is far more complex, colorful and interesting. The results presented herein mimic this in that the simple world of univariate analysis may not correctly predict the results of selection: it is only in the more complex world measured by genetic and phenotypic interactions among components, both within males and within female preferences, that multivariate evolution can be understood and predicted.

## Supplementary information

Supplemental material

## Data Availability

All data for the pedigree experiment are archived in Dryad: 10.5061/dryad.s4mw6m9h4.
