# Supplementary material for: The evolution of preferred male traits, female preference and the G matrix: “Toto, I’ve a feeling we’re not in Kansas anymore”
Source: Heredity (Edinb). 2025 Jan 12;134(3-4):162–74. doi: 10.1038/s41437-024-00744-8 (PMC11976997; doi:10.1038/s41437-024-00744-8)
Supplement: Supplementary file 1 — Supplemental material [file 41437_2024_744_MOESM1_ESM.docx]

**Supplementary Material**

**
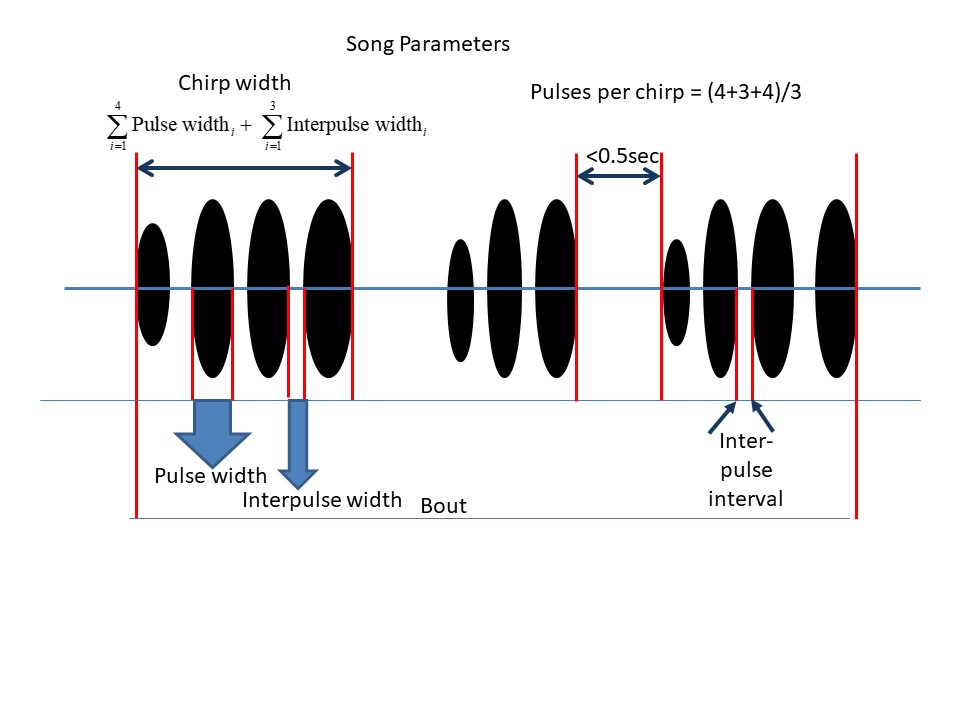
Figure S1:** Diagram of song components

**Analysis of Stabilizing Preference**

In stabilizing preference female preference declines as the difference between her most preferred trait value and the one encountered increases. In this case I used an iterative approach, first assigning a proposed “most preferred” value, say *X_P_*, and then calculating the absolute difference between this value and the male’s value. This is done for each male and preference then calculated as for direct preference by regressing *Y*, as defined above, on *X=*abs(Focal male trait-*X_P_*)/(sum of abs(Focal male trait- *X_P_*)+abs(Other male trait- *X_P_*)). The value of *X_P_* is varied, preference calculated for each value and plotted against the correlation of *Y* on *X* (Roff and Fairbairn 2017). If preference is stabilizing the correlation coefficient *r* will show a humped shaped relationship with *X_P_*. Significant stabilizing preference requires that the regression is negative (hence *r* is negative) and statistically significant at the value of *X_P_* at which the -*r* is maximal. Simulation analysis suggests that a significant level of 5% is weak evidence of stabilizing preference but a *P* value of 1% or less is strong support (Roff and Fairbairn 2017).

Theory is not a good guide to whether differences in stabilizing preference should be observed between the R and S lines. Therefore, I tested for differences between lines without an a priori prediction on the direction of change that might occur. To test for differences in detected stabilizing preference I used the following randomization test. First, I determined the most preferred male trait value for each line separately. Next I calculated the absolute difference between the two estimates, *d_obs_*. I then randomly assigned individuals to the S or R populations and reran the stabilization testing protocol, determining the two preferred male trait values, thence the absolute difference between them, *d_i_*. This process was repeated *n* times giving *n* values of *d_i_*.*.* The probability of observing a *d_i_* greater than or equal to *d*_obs_ is estimated as {(Number of *d_i_* ≥ *d_obs_* )+1}/(n+1). The “extra” 1 is required because the observed value is itself a sample and thus must be counted (Roff 2006). If the true probability is close to 0.05 then a sample size of 1,000 or more may be necessary to take into account the standard error of the estimated probability (Roff 2006). Because of the time required to run such a large number of runs, in the present analysis I used a sequential approach, first running 100 randomizations and increasing the number only if the estimated probability was less than 0.20 since the 95% confidence range is $0.2\pm2\sqrt{0.2*0.8/101}=0.2\pm0.08$, which excludes 0.05.

REFERENCE: Roff DA (2006). Introduction to Computer-Intensive Methods of Data Analysis in Biology. Cambridge University Press: Cambridge.

**Figure S2**: Distribution of PC scores for male song components. Traits are arranged according to ranking of PC1.

**Figure S3.** Plots of four traits showing stabilizing preference. Dashed line shows value of the correlation at which *P*=0.05 for the combined data set. For a detailed explanation of the statistical method see section above labelled “Analysis of Stabilizing Preference” and Roff DA, Fairbairn DJ, Prokuda A (2017). A new method for statistical detection of directional and stabilizing mating preference. Behav Ecol 28(3): 934-942.

**Figure S4:** Correspondence between the genetic and phenotypic correlations in the song components. Dotted line shows 1:1 relationship. Solid line shows regression of genetic correlation on phenotypic correlation (*r*_g_=-0.082+0.853*r*_p_, *P*<0.0001)

**Figure S5:** Comparison of male traits in generations 3 and 11 that showed a significant generation by line interaction.
